# Moderate grazing increased alpine meadow soils bacterial abundance and diversity index on the Tibetan Plateau

**DOI:** 10.1002/ece3.6563

**Published:** 2020-07-16

**Authors:** Yangong Du, Xun Ke, Licong Dai, Guangmin Cao, Huakun Zhou, Xiaowei Guo

**Affiliations:** ^1^ Qinghai Provincial Key Laboratory of Restoration Ecology for Cold Region Northwest Institute of Plateau Biology Chinese Academy of Sciences Xining China; ^2^ University of Chinese Academy of Science Beijing China

**Keywords:** alpine meadow, bacterial genes richness, diversity index, grazing intensity

## Abstract

The response of grassland soil bacterial community characteristics to different grazing intensities is central ecological topics. However, the underlying mechanisms between bacterial abundance, diversity index, and grazing intensity remain unclear. We measured alpine meadow soil bacterial gene richness and diversity index under four grazing intensities using 16S rDNA sequence analysis on the Tibetan Plateau. The results suggest that extreme grazing significantly decreased alpine meadow both bacterial gene abundance and diversity index (*p* < .05). The lowest operational taxonomic unit numbers were 3,012 ± 447 copies under heavy grazing in the growing season. It was significantly lower than heavy grazing with approximately 3,958 ± 119 copies (*p* < .05). The Shannon index for medium and high grazing grassland bacterial diversity was slightly higher than for light grazing in the growing season. Furthermore, the lowest index was approximately 9.20 ± 0.50 for extreme grazing of grassland in the growing season. The average bacterial gene abundance and diversity index in the dormancy period were slightly higher than that in the growing season. Soil bulk density, pH, ammonium, and nitrate nitrogen were the main positive factors driving grazed grassland bacterial communities. Our study provides insight into the response of alpine meadows to grazing intensity, demonstrating that moderate grazing increases bacterial community diversity in grazed grasslands.

## Graphical abstract

Extreme grazing steeply decreased alpine meadow bacterial genes abundance and diversity index. Shannon indexes of medium and high grazing grassland bacterial diversity were slightly higher than light grazing in growing season. Our study provides insight into alpine meadow bacterial response to grazing intensity, and moderate grazing helps increase grazing grasslands bacterial community.

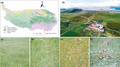

## 1 INTRODUCTION

Global grasslands have been suffering from degradation owing to climate change and long‐term overgrazing (Babel, Biermann, & Coners, 2014; Che et al., 2018). Livestock grazing is considered to be the most important land‐use activity for grasslands (Akiyama, Yan, & Yagi, 2010; Du, Ke, Guo, Cao, & Zhou, 2019). Moreover, grassland degradation drastically decreases the soil organic carbon and total nitrogen contents (Babel et al., 2014; Du, Ke, et al., 2019). Microbial abundance has an indirect impact on the grassland nitrogen and carbon turnover process (Chu et al., 2016; Hu, Nie, et al., 2019). There has been a growing interest in relating grassland bacterial community and abundance to grazing intensity.

Gene copy numbers were widely used to indicate microbial abundance (Batten, Scow, & Espel, 2008; Chu et al., 2016; Hu, Nie, et al., 2019). The 16S rDNA gene copy numbers of wetlands were much smaller than upland grassland soils (Park et al., 2012). The richness in grassland soil microbes was much higher with nitrate nitrogen fertilization than control grasslands (Song et al., 2018). Microbial abundance declined significantly when permanent grasslands were converted to bare fallow (Hirsch, Jhurreea, & Williams, 2017; Zhong, Yan, Wang, Wang, & Shangguan, 2018). Heavy grazing significantly reduces soil bacterial diversity indices in steppe grasslands (Qu et al., 2018). Bacterial abundance was significantly higher than the abundance of actinomycetes and fungi in grazed grasslands on the Tibetan Plateau (Ma, Shao, & Zhang, 2013). Moderate grazing significantly increased ammonia‐oxidizing archaea (AOA) gene abundance, but high grazing intensity decreased its abundance (Du, Ke, et al., 2019). The relative abundance of nitrifying bacterial functional genes was enhanced by moderate grazing (Ma et al., 2019). Alpine steppe microbial communities differed significantly with varying degrees of degradation (Zhou et al., 2019). However, it was also reported that there were no significant differences in microbial alpha diversity among different degraded alpine steppes (Jing, Sanders, Shi, Chu, & He, 2015; Zhou et al., 2019). Meta‐analysis results indicate that soil microbial biomass has a significant positive association with soil carbon, nitrogen, and water content (Zhan et al., 2020). Furthermore, total nitrogen and pH were also the main factors influencing the bacterial community and biomass of grazed grasslands (Qu et al., 2018).

The Tibetan Plateau extends for more than 2.5 million km^2^, is known as the “the Third Pole” of the earth, and is sensitive to global climate change (Chen et al., 2017). Alpine meadows are the main vegetation type covering 35% of the Tibetan Plateau (Jing et al., 2015). Grasslands have undergone intensive degradation due to long‐term overgrazing and an increase in animal production demands on the Tibetan Plateau (Du, Ke, et al., 2019; Qiu, 2016). Some types of the sedge family pasture have vanished from high grazing grassland; these pastures made livestock stronger and fatter (Qiu, 2016). The bacterial community structure of grasslands is distinct between surface and subsurface soil layers, strongly correlated with total carbon and soil physical characteristic on the Tibetan Plateau (Chu et al., 2016). Soil microbial activity is dependent on organic carbon substrate and temperature in alpine meadows (Du, Shu, Guo, & Zhu, 2019; Kato, Toyoda, Yoshida, Tang, & Wada, 2013). However, little is known on the characteristics of grassland soil bacterial biomass and diversity in response to different grazing intensities.

Understanding of these patterns would provide robust information for alpine meadow soil biological processes under different grazing intensities. We hypothesized that the grassland soil bacterial abundance and diversity index would decrease with increasing grazing intensity. Bacterial abundance and diversity index were higher in the growing season than the dormancy period.

## 2 MATERIALS AND METHOD

### 2.1 Site description

**FIGURE 1:**
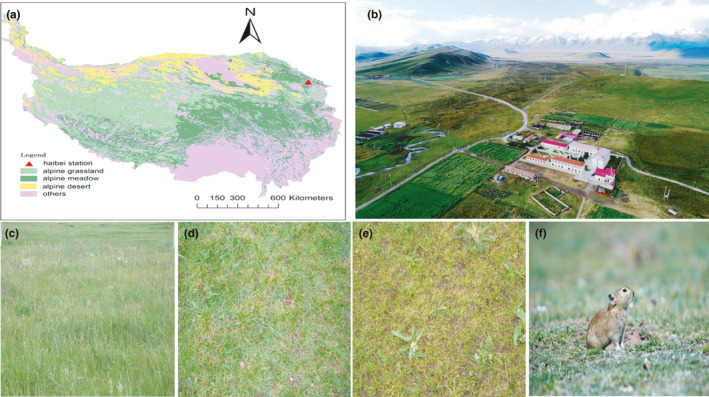
Geographical location of Haibei station and its landscape of four grazing grasslands (a means the main vegetation on the Tibetan Plateau and location of Haibei station; b showed the views of Haibei Station in the growing season by a drone; and c‐f indicated the vegetation growth and its main species at 4, 8, 12, and 16 sheep/ha)

This manipulation was carried out at Qinghai Haibei alpine grassland ecosystem national observation and research station ((37°32′N, 101°15′E, 3,280 m, Figure 1a). Annual precipitation and air temperature averaged at 560 mm and −1.7°C. The soil is classified as Mat‐Gryic Cambisols in alpine meadow, and it is rich in organic matter (Table 1).

**TABLE 1:** Soil basic characteristic under different grazing intensities in alpine meadow

Grazing intensity	SOC %	TN %	${\text{NH}}_{4}^{+}$ mg/kg	${\text{NO}}_{3}^{‐}$ mg/kg	pH	Bulk density g/cm^3^
Light grazing	6.87	0.83	11.4	6.5	7.5	0.75
Medium grazing	6.37	0.72	17.5	9.2	7.7	0.58
High grazing	4.91	0.65	19.6	10.5	7.7	0.54
Extreme grazing	4.16	0.47	12.3	6.8	7.9	1.01

*Kobresia humilis* and *K. pymaea* meadow is the main vegetation type in this region and on the Tibetan Plateau (Figure 1). It was fenced and used as winter grazing pasture by Tibetan sheep and yaks from the beginning of October to the next end of April (dormancy period). Growing season lasted approximately 5 months from May to late September. This plant community includes more than 30 species per m^2^. The main species were *K. humilis, K. pygmaea, Festuca ovina, F. rubra, Stipa aliena, Saussurea superba, Helictotrichon tibetica, Leontopodium nanum, Poa crymophila, and Potentilla saundersiana*.

### 2.2 Experimental design

This study aimed to investigate the response of the grassland bacterial community to different grazing intensities on the Tibetan Plateau. Four grasslands with different surface landscapes were selected as the focus of this study (Figure 1b). These grasslands suffered from different grazing intensities because of the family contract responsibility system since the 1980s (Table 1). These intensities consisted of 4 sheep/ha (light grazing, Figure 1c), 8 sheep/ha (medium grazing, Figure 1d), 12 sheep/ha (heavy grazing, Figure 1e), and 16 sheep/ha (extreme grazing, Figure 1f).

Four types of grassland soils were sampled using a 5 cm diameter soil core auger in July (growing season, Gro) and December (dormancy season, Dor) 2017 with three replicates. Soils of 24 copies were maintained at 4°C by an ice box during transport from the field to the laboratory. Then, soils were passed through a 2 mm sieve, while some of these samples were still frozen at −10°C for microbial analyses. The remaining soil samples were naturally air dried for physical and chemical analyses.

### 2.3 Soil sample sequencing

#### 2.3.1 Extraction of genomic DNA and amplicon generation

Total genomic DNA from soil samples was extracted using the cetyl trimethylammonium bromide/sodium dodecyl sulfate (CTAB/SDS) method. DNA concentration and purity were monitored on 1% agarose gels. Based on the concentration, DNA samples were diluted to 1 ng/μl using sterile water. A 16S rDNA genes of distinct regions (16SV4/16SV3/16SV3‐V4/16SV4‐V5, Arc V4) were amplified using specific primers (e.g., 16S V4: 515F‐806R) with the barcode. All polymerase chain reactions (PCR) were carried out with Phusion^®^ High‐Fidelity PCR Master Mix (New England Biolabs).

#### 2.3.2 PCR product quantification, qualification, mixing, and purification

The same volume of 1X loading buffer (containing SYB green) with PCR products and electrophoresis on 2% agarose gel for detection were mixed. Samples with a bright main strip between 400 and 450 bp were chosen for further experiments. PCR products were mixed in equidensity ratios and purified with a Qiagen Gel Extraction Kit (Qiagen, Germany).

#### 2.3.3 Library preparation and sequencing

Sequencing libraries were generated using TruSeq^®^ DNA PCR‐free sample preparation kit (Illumina, USA) following the manufacturer's recommendations, and index codes were added. The library quality was assessed on the Qubit@ 2.0 Fluorometer (Thermo Scientific) and Agilent Bioanalyzer 2100 system. The library was sequenced on an IlluminaHiSeq2500 platform, and 250 bp paired‐end reads were generated.

### 2.4 Data Analysis

#### 2.4.1 Operational taxonomic units' production, data normalization, and cluster analysis

Sequence analysis was performed using Uparse software (Uparse v7.0.1001, http://drive5.com/uparse/). Sequences with ≥97% similarity were assigned to the same operational taxonomic units (OTUs). Representative sequences for each OTU were screened for further annotation. OTU abundance information was normalized using a standard sequence number corresponding to the sample with the least sequences. Subsequent analysis of alpha diversity and beta diversity was performed based on the normalized output data.

The Shannon index was calculated (http://www.mothur.org/wiki/Shannon), and applied to reduce the dimensions of the original variables using the FactoMineR package and ggplot2 package in R software (v2.15.3).

### 2.5 Statistical analyses

One‐way ANOVA was conducted to analyze the effects of grazing intensities on OUT numbers and Shannon's diversity index in alpine meadow at significant level of *p* < .05 (SPSS 19). Canonical correlation analysis (CCA) was conducted between genes abundance and soil chemical properties in different grazing alpine meadows using RGCCA package in R (v2.15.3). Monte Carlo test was carried out to test the factor interpretation rate affecting microbial community using MCMC package in R (v2.15.3).

## 3 RESULT

### 3.1 Comparative analysis of OTU numbers under different grazing intensities

**FIGURE 2:**
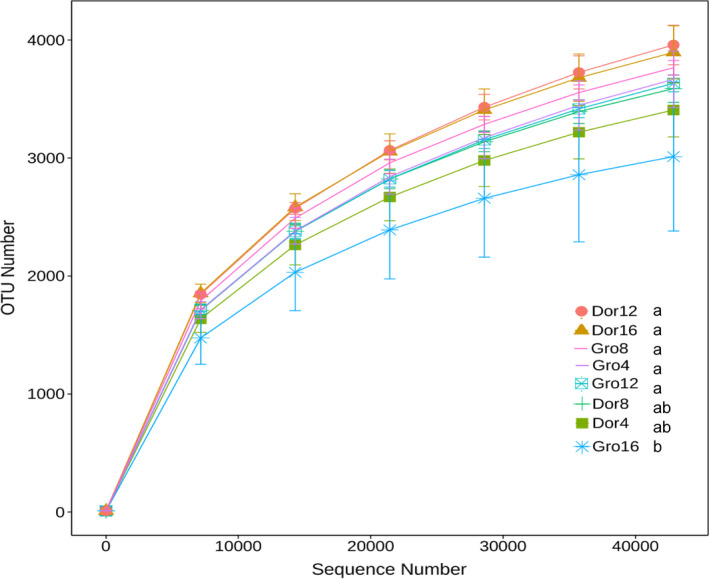
OTU numbers variation under different grazing intensity during growing and dormancy season under four grazing intensities (Dor 4, 8, 12, and 16 stood for the result at grazing intensity of 4, 8, 12, and 16 sheep/ha during dormancy period. Meanwhile, Gro indicated growing season). Different letters meant there was significant difference among groups (*p* < .05)

The OTU numbers provide an indication of microbial abundance in grassland soils. OTU was slightly higher (approximately 5.49%) in the dormancy season (3,712 ± 89) than in the growing season (3,519 ± 136) across all plots (Figure 2). Alpine meadow soil bacterial gene OTU increased steeply with the gene sequence number. The highest and lowest OTU was 3,958 ± 119 and 3,012 ± 447 copies for heavy grazing in the dormancy season and extensive grazing in the growing season, respectively (*p* < .05). The growing season OTU in descending order is medium grazing > light grazing > heavy grazing > extreme grazing grasslands (*p* < .05).

**FIGURE 3:**
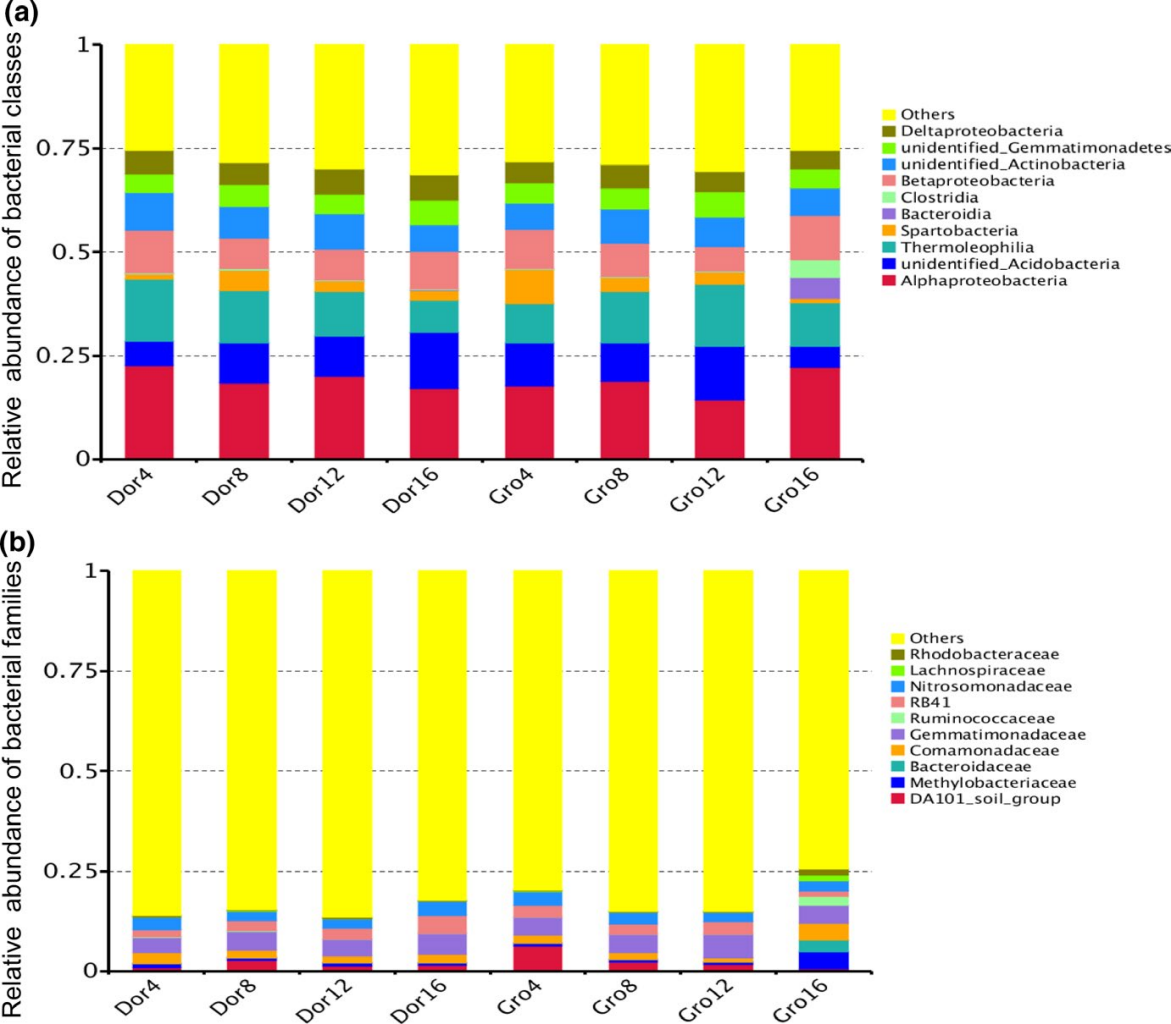
Bacterial classes (a) and the families (b) in the four grazing intensities during growing and dormancy season under four grazing intensities (Dor 4, 8, 12, and 16 stood for the result at grazing intensity of 4, 8, 12, and 16 sheep/ha during dormancy period. Meanwhile, Gro indicated growing season)

The main ten types of bacterial classes were identified, of which the fore three classes occupied principal section approximately 40.18 ± 2.09% (from 37.53% in Gro 4 to 43.47% in Dro 4). There classes were *Alphaproteobacteria*, u*nidentified Acidobacteria*, and *Thermoleophilia*. These relative abundances in medium and heavy grazing intensity were significantly higher than that in light and extreme grazing intensity during the growing season (Figure 3). With grazing intensity, these abundances gradually decreased during the dormancy period. Main identified and others bacterial families were approximately 17.05% and 82.95%, separately. The principle families included *Gemmatimonadaceae, Nitrosomonadaceae, RB41, Comamonadaceae,* and *DA101‐soil‐group,* approximately 4.70%, 2.79%, 2.69%, 2.16%, and 2.11%, separately. Gene abundances of *Gemmatimonadaceae* also indicated much higher in medium and heavy grazing intensity than that in light and extreme grazing intensity during the growing season. However, it was highest in the extreme grazing during the dormancy period (Figure 3).

### 3.2 Shannon's diversity index variation with increasing grazing intensity

**FIGURE 4:**
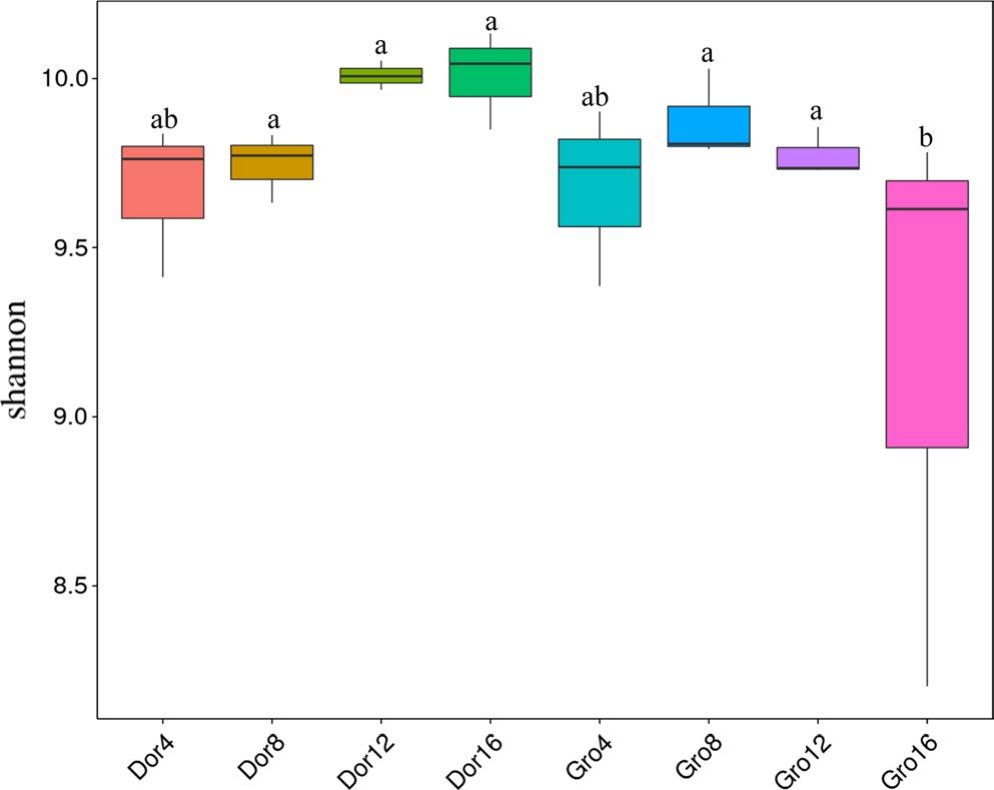
Shannon's diversity index variation of bacterial genes under different grazing intensity during growing and dormancy season under four grazing intensities (Dor 4, 8, 12, and 16 stood for the result at grazing intensity of 4, 8, 12, and 16 sheep/ha during dormancy period. Meanwhile, Gro indicated growing season). Different letters meant there was significant difference among groups (*p* < .05)

The Shannon index was used to verify the genetic diversity of each grazed alpine meadow. The average bacterial gene diversity index in the dormancy period was slightly higher (9.86 ± 0.06) than in the growing season (9.63 ± 0.05). With grazing intensity increasing, the Shannon index during the dormancy period for grasslands increased gradually. Medium and high grazing increased the grassland bacterial diversity index slightly compared with light grazing in the growing season (Figure 4). The Shannon index was at its lowest (approximately 9.20 ± 0.50) in the extreme grazed grassland in the growing season. The highest index was approximately 10.01 ± 0.03 in extreme grazed grassland during the dormancy period (Figure 4).

### 3.3 Canonical correlation analysis effect of soil environmental factors on bacterial gene characteristics

**FIGURE 5:**
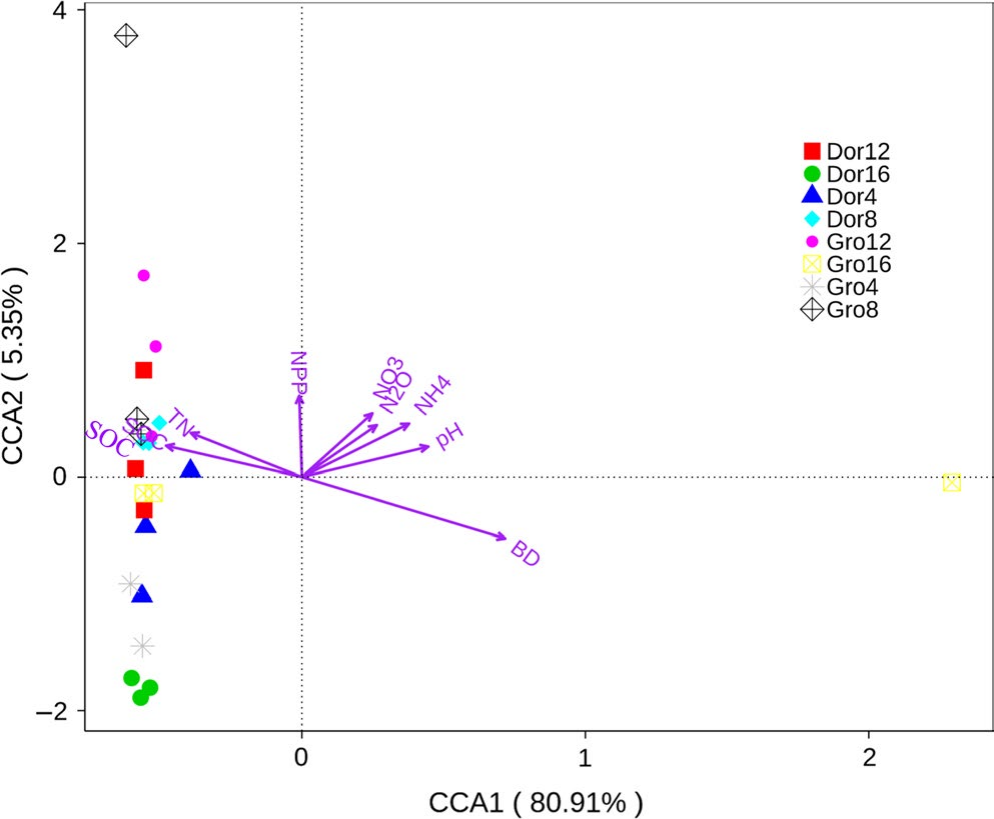
Canonical correlation analysis results between genes abundance and soil chemical properties in different grazing alpine meadows. Note, BD meant soil bulk density, and NH_4_ and NO_3_ meant soil ammonium nitrogen and nitrate content. N_2_O meant soil nitrous oxide emission rates. NPP stood for grassland net primary production. TN and SOC meant soil total nitrogen and soil organic carbon content

The CCA method is considered to be a general method for multivariate factors and bacterial gene abundance correlation statistical analysis. The cumulative explanatory variance reached 86.26%, and soil physical factors of the CCA1 axis play a vital role on gene abundance in alpine meadows. Soil bulk density, pH, ammonium nitrogen, and nitrate nitrogen were the main positive factors for grazing grassland gene abundance (Figure 5). Monte Carlo test indicated pH and bulk density significantly affected microbial classes community (*p* < .001, *r* = 0.29) and families community (*p* < .05, *r* = 0.24).

## 4 DISCUSSION

Global grasslands cover more than 25% of the world's land surface area (Asner, Elmore, & Olander, 2004). Grassland degradation becomes a major global problem (Hirsch et al., 2017), global warming and overgrazing being the main stressors causing grassland degradation (Liu et al., 2018). Heavy grazing significantly reduces grassland plant coverage and increased bare land area (Du, Ke, et al., 2019). Microbes played a vital role in nitrogen transformation and organic carbon decomposition (Park et al., 2012). The resistance and resilience of grassland ecosystems to grazing may be estimated in relation to the microbial community (Hu, Nie, et al., 2019). In addition, domestic herbivore grazing significantly influences the grassland soil bacterial community (Qu et al., 2018). Direct sunlight on bare ground decreases soil water content due to accelerating soil water evaporation, further constraining soil microbial richness (Zhan et al., 2020).

This study observed that extreme grazing intensity dramatically decreased alpine meadow soil bacterial richness and diversity index during the growing season. The most plausible explanation for this outcome is that alpine meadow soils suffer from oligotrophic inorganic N and organic carbon nutrition pool sizes (Chu et al., 2016; Hu, Liu, et al., 2019). Moreover, long‐term extreme grazing significantly decreases alpine meadow soil ammonium, nitrate nitrogen, and organic carbon significantly (Du, Zhou, Guo, & Cao, 2019; Zhang, Dong, & Gao, 2017). Total carbon was the best predictor for grassland soil microbial community distribution across the Tibetan Plateau (Chu et al., 2016).

Our results indicate that medium grazing significantly increased soil bacterial richness and diversity index compared with extreme grazing in the growing season. This is because soil quality is significantly increased by moderate grazing across China's grasslands, and soil quality was positively related to soil microbe biomass (Qu et al., 2018; Zhan et al., 2020). Furthermore, medium grazing promoted soil water‐holding capacity and litter biomass, both benefit to microorganism's utilization (Che et al., 2018; Guo et al., 2020). Similar results also have been found in previous studies, where moderate grazing has been observed to increase soil bacterial biomass and diversity in limestone and alpine grasslands (Amezaga, Mendarte, & Albizu, 2009; Zhao & Yu, 2017). Both the grassland bacterial richness and diversity index were slightly higher during the dormancy period than the growing season, with no significant differences. Grazing activity decreased the bacterial richness and diversity index in the growing season. This study analyzed soil genes abundance and diversity index, and these results did not represent bacterial activity (Che et al., 2018). Microbial nitrification was detected even in frozen soils by the ^15^N isotope dilution method in an alpine meadow (Hu, Nie, et al., 2019; Kato et al., 2013). Nitrogen‐cycling genes of nirS were negatively correlated with soil inorganic nitrogen in alpine meadows (Che et al., 2018). The grassland microbial community was altered by the deposited aerophile soils (Park et al., 2012). In this study, grasslands pH and bulk density significantly influenced microbial classes community by Monte Carlo test. Soils pH took a vital role in driving soil microorganism's communities (Du, Shu, et al., 2019; Hu, Nie, et al., 2019). Soils bulk affected grasslands soil porosity and oxygen contents, and it is positive correlated with soil bacterial richness on the Tibetan Plateau (Che et al., 2018; Chu et al., 2016).

These findings in this study help improve our understanding of the response of alpine meadow soil bacterial communities with different grazing intensities. Moderate grazing was suitable for the sustainable development of alpine meadows due to increasing both soil bacterial richness and diversity index.

## CONFLICT OF INTEREST

None declared.

## AUTHOR CONTRIBUTIONS

**Yangong Du:** Formal analysis (lead). **Xun Ke:** Data curation (lead). **Licong Dai:** Data curation (supporting). **Guangmin Cao:** Investigation (supporting). **Huakun Zhou:** Software (supporting). **Xiaowei Guo:** Writing–review and editing (supporting).

## Data Availability

Grassland soils bacterial diversity data in this paper were deposited in dryad https://doi.org/10.5061/dryad.cfxpnvx36.
